# Adoption of Telemedicine in Low-Resource Settings Through the Lens of Frugal Innovation: Protocol for a Systematic Review

**DOI:** 10.2196/83311

**Published:** 2026-07-03

**Authors:** Christopher Adlung, Pouria Paridar, Caroline Figueroa, Christa Niehot, Nikita Kanumoory Mandyam, Cees van Beers, Saba Hinrichs-Krapels

**Affiliations:** 1 Faculty of Technology, Policy, and Management, Delft University of Technology, Building 31, Jaffalaan 5, Delft, 2628 BX, The Netherlands, 31 015 278 9111; 2 Medical Library, Erasmus Medical Center Rotterdam, Rotterdam, The Netherlands; 3 Fuqua School of Business, Duke University, Durham, NC, United States

**Keywords:** telemedicine, frugal innovation, adoption, low-resource settings, health care access

## Abstract

**Background:**

Access to health care, especially in low-resource settings, remains a critical challenge for marginalized and underserved populations. Telemedicine, delivering medical advice remotely, has the potential to improve access to health care by reducing geographic and economic barriers. Its adoption, however, especially in low-resource settings, remains limited. Frugal innovation, characterized by solutions developed under tremendous resource constraints and prioritizing affordability, accessibility, and simplicity, offers valuable insights for enhancing telemedicine adoption. Rooted in a sociocultural approach, it is particularly relevant in low-resource health systems where complex, intersectoral challenges occur. Despite this potential, insights from frugal innovation have not been compared, contrasted, or studied alongside telemedicine in a systematic way.

**Objective:**

Our review aims to examine how frugal innovation contributes to the adoption of telemedicine in low-resource settings, with a particular focus on identifying frugal innovation characteristics that facilitate telemedicine adoption.

**Methods:**

Our PRISMA (Preferred Reporting Items for Systematic Reviews and Meta-Analyses)-guided systematic review will examine case studies of adopted telemedicine applications in low-resource settings. Eligible studies need to demonstrate evidence of adoption and use in real-world health care settings. We will examine functions and features of telemedicine, along with frugal innovation characteristics, as demonstrated by each telemedicine application. The following databases were searched: MEDLINE ALL, Embase, Web of Science Core Collection, Cochrane Central Register of Controlled Trials, CINAHL, PsycInfo, Scopus, Dimensions, IEEE Xplore Digital Library, EconLit, the International HTA Database, LILACS (Latin American and Caribbean Literature on Health Sciences), the World Health Organization (WHO) Global Index Medicus, and the WHO Global Health Observatory.

**Results:**

Insights from our review will deepen the understanding of how health care technologies such as telemedicine can be positioned and configured to improve adoption in low-resource settings. Search results, delivered in February 2025, were followed by title and abstract screening in March 2025, full-text screening in April 2025, and final study selection and quality appraisal by November 2025. With dataset updates requested in December 2025, the data synthesis remains ongoing. The expected results will be published in 2026.

**Conclusions:**

This combined assessment will examine how frugal innovation can contribute to the adoption of telemedicine in low-resource settings. As a result, we expect that our findings will contribute to the development of frugal telemedicine as a sociotechnical artifact. The protocol is submitted to enhance transparency and rigor.

## Introduction

### Background

Access to health care on a global scale remains more a promise than a reality. Although international human rights frameworks affirm that everyone is entitled to a standard of living adequate for health and well-being [1], access to health care in high-resource settings vastly differs from access in low-resource settings.

Telemedicine, often viewed as a technology-driven solution, has emerged as a potential tool to expand access to health care beyond geographical limitations. According to the World Health Organization (WHO), telemedicine is defined as “the delivery of health care services, where distance is a critical factor, by all health care professionals using information and communications technologies for the exchange of valid information for diagnosis, treatment and prevention of disease and injuries, research and evaluation, and the continuing education of health care workers, with the aim of advancing the health of individuals and communities” [2]. In practice, this means that telemedicine involves the remote communication of medical advice through digital modalities such as synchronous video consultations or asynchronous messaging applications.

Despite this promising nature of telemedicine, its adoption is still challenged by various obstacles [3]. Barriers in high-income settings mostly encompass confidentiality issues [4], privacy and legal concerns [5], resistance to change [6], workflow issues [7], and interoperability challenges [8]. Here, the adoption of telemedicine is often facilitated by existing infrastructure, established health policies, and broad access to technology, as illustrated by the use of telepsychiatry [9].

Barriers in low-resource settings differ from this. Language barriers [10], understanding medical jargon [11], the need to share a single phone [12], unawareness of technology, technically challenged staff, poor design [13], high expectations of users [14], and implementation costs [15] remain key challenges. As a result, health care systems in low-resource settings are often hindered by infrastructural voids, financial restrictions, and sociocultural resistance.

The literature identifies these contextual barriers but lacks theory-driven insights into how innovation characteristics support adoption of telemedicine in low-resource settings. In particular, the mechanisms through which factors such as affordability, simplicity, adaptability, and cultural fit influence telemedicine adoption remain underexplored [16].

Addressing this gap is essential to position telemedicine as a meaningful solution for health care access.

Frugal innovation, with its emphasis on context-sensitive and resource-efficient solutions, provides a promising lens for this purpose. According to Bhatti et al. [17], frugal innovation is defined as “innovation under constraints” and as a conceptual model with a wide range of perspectives, including “market affordability constraints,” “resource constraints or scarcity,” and “institutional voids or complexities.”

Existing research on frugal innovation, outside the scope of telemedicine, has revealed intricate findings which could offer insights for the adoption of telemedicine. For instance, the more complex an innovation or the setting in which it is introduced becomes, the less likely it is to be successfully adopted [18-21]. This may provide a hint as to how complexity reduction, as a core aspect of frugal innovation, could offer solutions in the interest of adoption, especially for advanced technology concepts such as telemedicine [22].

Specifically for telemedicine, few researchers, such as Li et al. [23], demonstrate how frugal approaches can effectively enhance telemedicine and broaden health care access for underprivileged populations in low-resource settings by using technology affordances in case studies such as free telemedicine camps. These “camps” use frugal innovation in mobile health units such as “Hospitals on Wheels.” The research by Li et al. [23] reveals that patient satisfaction is influenced by aligning the “media richness” of telemedicine with factors such as diagnostic complexity and health care needs, emphasizing how effectively implemented frugal solutions can address diverse medical requirements in a cost-efficient manner. This indicates that the reduction of technological complexity, in combination with a socially oriented health care need, improves adoption and access in favor of telemedicine.

However, a systematic evidence base on how frugal innovation characteristics influence telemedicine adoption in low-resource settings remains missing. Therefore, our study will investigate the following question: How do frugal innovation characteristics contribute to the adoption of telemedicine solutions in low-resource settings?

Accordingly, we will adopt a sociotechnical and multiactor systems perspective in this study. Specifically, we aim to synthesize evidence of case studies in diverse low-resource contexts to assess how frugal innovation characteristics, such as affordability, accessibility, or simplicity, interact with telemedicine technologies. While this contributes to the conceptualization of frugal telemedicine, our review will further examine which frugal innovation characteristics will lead to the adoption of telemedicine and how they do so by exploring the environmental, technological, and system-level factors shaping the adoption process. Where available, our study will further identify relevant variables that may support advanced analytical approaches, such as latent class analysis, to deepen understanding of adoption patterns in resource-constrained health care settings.

### Foundational Concepts and Definitions

#### Telemedicine

In our review, we follow the definition of the WHO, viewing telemedicine as “the delivery of health care services, where distance is a critical factor, by all health care professionals using information and communication technologies [24] for the exchange of valid information for diagnosis, treatment and prevention of disease and injuries, research and evaluation, and for the continuing education of health care providers, all in the interests of advancing the health of individuals and their communities” [24]. This will include all modalities, including synchronous and asynchronous ways of communication. A practical example of telemedicine as a health technology is presented in telemedicine camps connecting underprivileged patients with physicians in remote cities and countries [23].

#### Frugal Innovation

Frugal innovation has been widely discussed in the literature as an approach to delivering “more with less” in resource-constrained environments [25]. As a solution, frugal innovation typically emphasizes affordability [26], simplicity, essential functions, and high consumer benefits. We view frugal innovation as a resource-scarce solution designed and implemented despite tremendous financial, technological, material, or other resource constraints [27].

Beyond well-known examples such as the Aravind Eye Care System [28], other applications include low-cost portable diagnostic devices, SMS text messaging-based health interventions, and simplified teleconsultation platforms designed for low-bandwidth environments. These examples illustrate how frugal innovation prioritizes core functionality and accessibility over technological sophistication. However, despite growing needs and interest, the adoption of frugal innovation principles within telemedicine remains underexplored.

#### Adoption

According to Sussman et al. [29], adoption is seen as “a decision of an organization or community to commit to and initiate an evidence-based intervention.” Proctor et al. [30] view adoption as the intention, initial decision, or action to try or use an innovation. On the basis of these definitions, we define adoption as an act of an individual or a group to choose and to use an innovation as a beneficiary or end user. As an example, an emergency department staff member uses a telehealth solution in their clinical practice. While many articles discuss adoption, we critically reflect on whether a device is truly used or simply reflects its adoption potential. Therefore, our study grounds the concept of adoption in implementation science research by distinguishing between initial acceptance and actual use as an indicator of successful implementation.

#### Low-Resource Setting

Following the definition by van Zyl et al. [31], we understand low-resource settings as environments characterized by financial insecurity, limited health services, underdeveloped infrastructure, restricted social and human resources, gaps in knowledge, cultural and geographic constraints, and research challenges, all of which collectively hinder the accessibility of health care.

### Research Gap

While telemedicine and frugal innovation have both been widely studied, the intersection between these concepts, specifically in relation to adoption in low-resource settings, remains underexplored. Existing studies vaguely outline which functions and features frugal innovation principles support and how this translates into adoption and use. We argue that most articles discuss the concept of adoption but most likely mean adoption potential rather than actual adoption. Understanding the contribution of frugal innovation between adoption potential and actual adoption of telemedicine solutions in low-resource settings remains pending.

### Objectives

We aim to synthesize and critically evaluate the existing literature on telemedicine and frugal innovation in the context of adoption in low-resource settings. We hereby focus on qualitative data. The primary research question (RQ) guiding our review is as follows:

- RQ 1: How do frugal innovation characteristics contribute to the adoption of telemedicine solutions in low-resource settings?

The following subresearch questions will help to address the primary RQ:

- RQ 2: What are the frugal innovation characteristics related to telemedicine application?
- RQ 3: What are the functions and features of telemedicine in low-resource settings?
- RQ 4: What environmental factors facilitate the adoption of telemedicine?
- RQ 5: Are there additional factors that influence the adoption of innovations in low-resource settings?
- RQ 6: If available, which variables can be identified for latent class analysis?

## Methods

### Review Strategy

Our review follows a structured process in accordance with the PRISMA (Preferred Reporting Items for Systematic Reviews and Meta-Analyses) guidelines. Details of the PRISMA checklist are provided in Checklist 1.

First, we will perform a database search using the concepts of telemedicine and frugal innovation from the literature.

Second, the screening process will encompass three stages: (1) initial screening, (2) full-text screening, and (3) final inclusion decision in the systematic review.

Third, data will be extracted from all eligible records, including characteristics of frugal innovation, functions of telemedicine, and features of telemedicine. Inclusion and exclusion criteria have been identified, guided by the Sample, Phenomenon of Interest, Design, Evaluation, Research type (SPIDER) framework.

Fourth, the methodological quality will be assessed via the Mixed Methods Appraisal Tool (MMAT).

Fifth, data synthesis will be conducted using a narrative synthesis approach.

### Database Search

Following the PRISMA guidelines [32], we will perform a systematic search across the following databases: MEDLINE ALL, Embase, Web of Science Core Collection, Cochrane Central Register of Controlled Trials, CINAHL, PsycInfo, Scopus, Dimensions, IEEE Xplore Digital Library, EconLit, the International HTA Database, LILACS (Latin American and Caribbean Literature on Health Sciences), WHO Global Index Medicus, and the WHO Global Health Observatory.

To sufficiently answer the RQs, our search strategy encompasses key concepts through a series of Boolean operators (“AND” and “OR”), related to telemedicine and frugal innovation. To ensure relevant studies are not missed, adoption as a terminology is excluded from the search string. Instead, each study will be manually assessed for its relevance to adoption. The search will be conducted without the application of date or geographic restrictions or limitations on peer-reviewed literature only. Study types will include empirical studies, PhD theses, gray literature, unindexed material, quality control studies, single case studies, multisite studies about telemedicine according to the telemedicine definition, and book chapters. Publication types will be filtered during screening to ensure relevance. The entire search strategy, being developed by an information specialist (CN) in cooperation with the lead author (CA), can be accessed in Multimedia Appendix 1.

### Screening Process

Screening will encompass a multistage process, involving (1) initial screening of titles, abstracts, and keywords; (2) full-text review for eligibility; and (3) final inclusion decision for the systematic review. Two independent researchers will perform a blinded screening to evaluate subject relevance for telemedicine and frugal innovation, with a third reviewer resolving conflicts. Full-text screening will be conducted by multiple researchers, with conflicts resolved through regular alignment and consensus meetings.

### Data Extraction

Data will be extracted from all records eligible based on characteristics of frugal innovation, functions of telemedicine, and features of telemedicine. Reviewer disagreements will be resolved through discussions, with consensus documented in the final data extraction table in Microsoft Excel. An assessment of adoption will be performed by interpretation by the researchers. Data will be extracted and managed using Covidence (Veritas Health Innovation Ltd), documented in a data extraction table. Table 1 outlines the data items to extract in accordance with the RQs.

**Table 1.** Data items to extract.

Relevant RQs^a^	Data items
RQ 1: How do frugal innovation characteristics contribute to the adoption of telemedicine solutions in low-resource settings?	Author information Year of publication Mixed Methods Appraisal Tool study type Study design information Country Technology in use Use cases of telemedicine Attributes of use cases of telemedicine Relationship variables between telemedicine and frugal innovation Adoption factors (barriers and facilitators)
RQ 2: What are the frugal innovation characteristics related to telemedicine application?	Characteristics of frugal innovation (eg, simplicity) Identified triggers for frugal innovation
RQ 3: What are the functions and features of telemedicine in low-resource settings?	Functions of telemedicine (eg, remote diagnosis) Features of telemedicine (eg, accessibility)
RQ 4: What environmental factors facilitate the adoption of telemedicine?	Technology-related adoption factors System-related adoption factors
RQ 5: Are there additional factors that influence the adoption of innovations in low-resource settings?	Adoption frameworks, theories, models, or guidelines used Identified triggers for frugal innovation (eg, profit vs problem driven)
RQ 6: If available, which variables can be identified for latent class analysis?	Categorical data points (ie, nominal or ordinal data points) Continuous data points (eg, demographics, health conditions)

a RQ: research question.

### Inclusion and Exclusion Criteria

Our inclusion and exclusion criteria for selected studies will be guided by the SPIDER framework, considering sample, phenomenon of interest, design, evaluation, and research type [33]. We will include case studies that have been implemented in low-resource settings, incorporating the concepts of telemedicine and frugal innovation.

Inclusion criteria can be found in Table 2.

Exclusion criteria can be found in Table 3.

**Table 2.** Inclusion criteria.

Priority	Criteria	Description
1	Document type	Empirical studies PhD theses Gray literature Unindexed material Quality control studies Single case studies Multisite studies about telemedicine according to the World Health Organization definition Book chapters
1	Languages	Inclusion of all languages
1	Publication date	No publication date restrictions
1	Telemedicine	Any concept falling within the definition of telemedicine will be considered, including but not limited to eHealth, telehealth, mobile health, ubiquitous health, telepsychiatry, virtual medicine, and telecare^a^
1	Frugal innovation	Studies will be included if they address characteristics related to frugal innovation, including but not limited to affordability, accessibility, and scalability

a The entire list of search terms can be found in Multimedia Appendix 1.

**Table 3.** Exclusion criteria.

Priority	Criteria	Description
1	Noneligible document types	Reviews Policy documents and guidelines (e.g., World Health Organization framework concepts) Commentaries Editorial working papers Posters Preprints (documents that have not been reviewed yet by a scientific audience) Conference papers for which only the abstract is available Letters Written forms of communication among editors Entire books
1	Unavailable full text	If no full text is available, the document will be excluded from our review
2	Subject incompatibility for telemedicine	Information support or management systems Interventions designed to disincentivize use behaviors without medical activities Medical interventions designed for nonhumans (e.g., dogs, cats, and horses) Electronic patient records that do not directly relate to medical activities performed over physical distance and time Population-based applications that have no medical activity performed or cover preventive health and lifestyle changes^a^
2	Subject incompatibility for frugal innovation	Studies that focus solely on low-resource settings without demonstrating frugal innovation characteristics, as defined for the intervention^b^
2	Missing adoption context	If telemedicine and frugal innovation are not used in the context of technology adoption
2	Geographical mismatch	The telemedicine application is adopted in a high-resource setting

a Example: financing application which does not involve the concept of clinical care.

b Example: studies in which authors label a telemedicine solution as frugal despite the absence of concrete characteristics such as affordability.

### Methodological Quality

We will assess the methodological quality of the included studies using the appropriate critical appraisal tools provided by MMAT [34]. This tool is specifically designed to assess the methodological quality of case studies included in systematic reviews, incorporating various study designs. The selection of the MMAT assessment will be based on the study design of each included study. Quality assessments will be conducted independently by 2 reviewers. Discrepancies will be resolved through consultation with a third reviewer.

### Data Synthesis

A narrative synthesis will ensure accessibility for nonexpert audiences by avoiding jargon, explaining terms as needed, and adhering to open-access standards. This narrative will be aligned with the RQs to ensure a clear connection between the synthesis and the overarching goals of the review. To ensure the accuracy and quality of translations, we will implement a standardized translation protocol.

### Ethical Considerations

Ethics approval is not required, as this study does not involve the collection of primary or clinical data. Review findings will be shared via peer-reviewed journal publications, conferences, stakeholder meetings, and online presentations.

## Results

The search results were delivered on February 27, 2025, followed by the initiation of title and abstract screening in early March 2025. Screening progressed throughout March 2025 with successive batches of article review and continued alignment between researchers, during which conflicts were identified and progressively addressed. Mid-March marked the resolution of several discrepancies and refinement of the screening approach and inclusion criteria. By the end of March 2025, the title and abstract screening phase was completed.

In early April 2025, additional conflicts were resolved and the screening process was finalized, with studies advancing to full-text review and the screening framework being further defined. Full-text screening was completed in April 2025. In November 2025, final study selection and quality appraisal were concluded, and in December 2025, a request was made to update the dataset. As of April 2026, the systematic review manuscript has not yet been submitted for peer review. While full-text screening has been completed, data synthesis and the formulation of results and conclusions remain in progress. The purpose of submitting this protocol for peer review is to enhance transparency and rigor in reporting.

## Discussion

### Anticipated Findings

In our review, we aim to explore how frugal innovation characteristics contribute to the adoption of telemedicine solutions in low-resource settings. While we understand this task as a contribution to conceptualizing frugal telemedicine, we also aim to focus on the perspective of how innovation characteristics can lead to better adoption. Here, we argue that adoption may not be considered a discrete, binary event but rather a processual perception among individuals and groups across different levels (i.e., individual, organizational, and system levels).

Although frugal innovation is frequently presented as a promising complement to telemedicine in resource-constrained settings, its role in adoption remains insufficiently theorized. Specifically, it is necessary to critically reflect on for whom, under what conditions, and at which levels frugal innovation characteristics support telemedicine adoption. Therefore, this review aims to move beyond descriptive adoption barriers and facilitators by examining the underlying mechanisms and contextual requirements through which frugal innovation characteristics may influence adoption in low-resource settings.

The findings are expected to inform future empirical research, supporting more context-sensitive approaches to the design and adoption of telemedicine interventions in low-resource settings.

### Limitations

Our review is not intended to generate causal claims but rather to clarify patterns, mechanisms, and knowledge gaps regarding the adoption of telemedicine in low-resource settings. The interdisciplinary nature of our study will likely result in heterogeneous findings, which may limit the identification of operationalizable and measurable outcomes. Consequently, a narrative synthesis is proposed, where findings will be interpreted in a context-sensitive manner. Relevant studies may not explicitly identify interventions as frugal innovation, which can introduce an interpretive element. We aim to mitigate this limitation through a comprehensive search strategy, using a wide range of relevant search terms, as well as independent screening and consensus-based alignment procedures.

Telemedicine adoption and frugal innovation require sensitivity from a sociocultural and technical standpoint. However, inconsistent reporting of these dimensions across studies may constrain the depth and interpretability of our synthesis. The review may also be subject to publication and language bias, as studies reporting unsuccessful or negative adoption experiences may be underrepresented.

## Supplementary material

10.2196/83311 Multimedia Appendix 1 Search strategy.

10.2196/83311 Checklist 1 PRISMA-P checklist.

## Abbreviations

LILACS: Latin American and Caribbean Literature on Health Sciences
MMAT: Mixed Methods Appraisal Tool
PRISMA: Preferred Reporting Items for Systematic Reviews and Meta-Analyses
RQ: research question
SPIDER: Sample, Phenomenon of Interest, Design, Evaluation, Research type
WHO: World Health Organization
