# Supplementary material for: Adoption of Telemedicine in Low-Resource Settings Through the Lens of Frugal Innovation: Protocol for a Systematic Review
Source: JMIR Res Protoc. 2026 Jul 3;15:e83311. doi: 10.2196/83311 (PMC13330645; doi:10.2196/83311)
Supplement: Multimedia Appendix 1 [file resprot-v15-e83311-s001.pdf]

Frugal innovations of telemedicine

Database searched	Platform	Years of coverage	Records	Records after duplicates removed
Medline ALL	Ovid	1946 - Present	1716	1699
Embase	Embase.com	1971 - Present	1667	323
Web of Science Core Collection*	Web of Knowledge	1975 - Present	2685	1210
Cochrane Central Register of Controlled Trials	Wiley	1992 - Present	191	13
CINAHL**	EBSCO	1982 - Present	607	176
PsycINFO	Ovid	1806 - Present	298	107
Scopus	Scopus.com	1823 - Present	3527	1113
Dimensions***	app.dimensions.ai/discover/publication		2618	1024
IEEE Xplore Digital Library	ieeexplore.ieee.org/search/advanced	1884 - present	324	303
EconLit	ProQuest	1886 - present	20	1
International HTA database	database.inahta.org/		11	9
Latin America and the Caribbean Literature on Health Sciences (LILACS)	lilacs.bvsalud.org/en/		147	144
**Total**			**13811**	**6122**

*Science Citation Index Expanded (1975-present) ; Social Sciences Citation Index (1975-present) ; Arts & Humanities Citation Index (1975-present) ; Conference Proceedings Citation Index- Science (1990-present) ; Conference Proceedings Citation Index- Social Science & Humanities (1990-present) ; Emerging Sources Citation Index (2005-present)

**Limited to Academic Journals

***Manually deleted book and book chapters

new references: 1585

Medline

(exp "Telemedicine"/ OR "Digital Technology"/ OR (telehospital* OR tele-hospital* OR e-health OR ehealth* OR mhealth* OR m-health* OR telehealth* OR tele-health* OR telemedic* OR tele-medic* OR telemonitor* OR tele-monitor* OR telecare OR tele-care OR tele-icu* OR tele-intensive-car* OR telephone-intervention* OR remote-consultat* OR telepresence* OR tele-presence* OR tele-referral* OR telereferral* OR teleconsultat* OR tele-consultat* OR teleradiol* OR tele-radiol* OR telepathol* OR tele-pathol* OR ((digital* OR remot* OR electronic* OR virtual* OR mobile) ADJ3 (health* OR intervention* OR consult* OR diagno* OR medicine*)) OR health-information-technolog* OR health-IoT OR h-IoT OR uhealth OR telerehabilitat* OR tele-rehabilitat* OR ((remot*)

ADJ3 (monitor*)) OR ((smart*) ADJ3 (health*)) OR ((health OR medical) ADJ2 (internet-of-things)) OR ((digital* OR remot* OR electronic* OR virtual* OR mobile) ADJ3 (distanc*) ADJ3 (practice*))).ab,ti,kf.) **AND** ((((inclusive* OR reverse* OR catalytic* OR lean OR cost* OR DIY OR sustain* OR grassroot* OR grass-root* OR bottom-up* OR bottomup* OR affordab* OR disruptive* OR lowcost*) ADJ3 (innovat*)) OR bootstrap* OR boot-strap* OR frugal* OR ((bottom* OR base) ADJ3 (pyramid* OR piramid*)) OR ((resource*) ADJ3 (constrain* OR scarc*)) OR bricoleur* OR bricolage* OR pro-poor* OR BoP OR jugaad or jugaar).ab,ti,kf.) **NOT** ("Editorial".pt. OR "Comment".pt. OR "Letter".pt. OR "News".pt. OR "Congress".pt. OR "Meeting Abstract".pt. OR (book* OR chapter* OR dissertation abstract*).pt.)

Embase

('telehealth'/exp OR 'telemedicine'/de OR 'telemonitoring'/exp OR 'digital health'/exp OR 'digital health technology'/exp OR 'digital health intervention'/exp OR 'electronic consultation'/exp OR 'mhealth'/exp OR 'mobile health'/exp OR 'mobile health application'/exp OR 'mobile technology'/exp OR 'telecare'/exp OR 'telehealth'/exp OR 'telemonitoring'/exp OR (telehospital* OR tele-hospital* OR e-health* OR ehealth* OR mhealth* OR m-health* OR telehealth* OR tele-health* OR telemedic* OR tele-medic* OR telemonitor* OR tele-monitor* OR telecare OR tele-care OR tele-icu* OR tele-intensive-car* OR telephone-intervention* OR telepresence* OR tele-presence* OR tele-referral* OR telereferral* OR teleconsultat* OR teleradiol* OR tele-radiol* OR ((digital* OR remot* OR electronic* OR virtual* OR mobile) NEAR/3 (health* OR intervention* OR consult* OR diagno* OR medicine*)) OR health-information-technolog* OR health-IoT OR h-IoT OR uhealth OR ((remot*) NEAR/3 (monitor*)) OR ((smart*) NEAR/3 (health*)) OR ((health OR medical) NEAR/2 (internet-of-things)) OR ((digital* OR remot* OR electronic* OR virtual* OR mobile) NEAR/3 (distanc*) NEAR/3 (practice*))):ab,ti,kw) **AND** ('bootstrapping'/exp OR (((inclusive* OR reverse* OR catalytic* OR lean OR cost* OR DIY OR sustain* OR grassroot* OR grass-root* OR bottom-up* OR bottomup* OR affordab* OR disruptive* OR lowcost*) NEAR/3 (innovat*)) OR bootstrap* OR boot-strap* OR frugal* OR ((bottom* OR base) NEAR/3 (pyramid* OR piramid*)) OR ((resource*) NEAR/3 (constrain* OR scarc*)) OR bricoleur* OR bricolage* OR pro-poor* OR BoP OR jugaad or Jugaaṛ):ab,ti,kw) **NOT** ([Conference Abstract]/lim OR 'editorial'/it OR 'letter'/it OR 'note'/it OR 'chapter'/it OR 'conference abstract'/it) NOT 'clinical trial'/it

Web of Science

TS=((telehospital* OR tele-hospital* OR e-health* OR ehealth* OR mhealth* OR m-health* OR telehealth* OR tele-health* OR telemedic* OR tele-medic* OR telemonitor* OR tele-monitor* OR telecare OR tele-care OR tele-icu* OR tele-intensive-car* OR telephone-intervention* OR telepresence* OR tele-presence* OR tele-referral* OR telereferral* OR teleconsultat* OR teleradiol* OR tele-radiol* OR ((digital* OR remot* OR electronic* OR virtual* OR mobile) NEAR/2 (health* OR intervention* OR consult* OR diagno* OR medicine*)) OR health-information-technolog* OR health-IoT OR h-IoT OR uhealth OR ((remot*) NEAR/2 (monitor*)) OR ((smart*) NEAR/2 (health*)) OR ((health OR medical) NEAR/2 (internet-of-things)) OR ((digital* OR remot* OR electronic* OR virtual* OR mobile) NEAR/2 (distanc*) NEAR/3 (practice*))) AND (((inclusive* OR reverse* OR catalytic* OR lean OR cost* OR DIY OR sustain* OR grassroot* OR grass-root* OR bottom-up* OR bottomup* OR affordab* OR disruptive* OR lowcost*) NEAR/2 (innovat*)) OR bootstrap* OR boot-strap* OR frugal* OR ((bottom* OR base) NEAR/2 (pyramid* OR piramid*)) OR ((resource*) NEAR/2 (constrain* OR

scarc*)) OR bricoleur* OR bricolage* OR pro-poor* OR BoP OR jugaad or Jugaaṛ)) **NOT** DT=(Meeting Abstract OR Meeting Summary)

Cochrane CENTRAL

((telehospital* OR tele NEXT/1 hospital* OR e NEXT/1 health OR ehealth* OR mhealth* OR m NEXT/1 health* OR telehealth* OR tele NEXT/1 health OR telemedic* OR tele NEXT/1 medic* OR telemonitor* OR tele NEXT/1 monitor* OR telecare OR tele NEXT/1 care OR tele NEXT/1 icu* OR tele NEXT/1 intensive NEXT/1 car* OR telephone NEXT/1 intervention* OR telepresence* OR tele NEXT/1 presence* OR tele NEXT/1 referral* OR telereferral* OR teleconsultat* OR teleradiol* OR tele-radiol* OR ((digital* OR remot* OR electronic* OR virtual* OR mobile) NEAR/3 (health* OR intervention* OR consult* OR diagno* OR medicine*)) OR health NEXT/1 information NEXT/1 technolog* OR health NEXT/1 IoT OR h NEXT/1 IoT OR uhealth OR ((remot*) NEAR/3 (monitor*)) OR ((smart*) NEAR/3 (health*)) OR ((health OR medical) NEAR/2 (internet NEXT/1 of NEXT/1 things)) OR ((digital* OR remot* OR electronic* OR virtual* OR mobile) NEAR/3 (distanc*) NEAR/3 (practice*))):ab,ti,kw) AND ((((inclusive* OR reverse* OR catalytic* OR lean OR cost* OR DIY OR sustain* OR grassroot* OR grass NEXT/1 root* OR bottom NEXT/1 up* OR bottomup* OR affordab* OR disruptive* OR low NEXT/1 cost* OR lowcost*) NEAR/3 (innovat*)) OR bootstrap* OR boot-strap* OR frugal* OR ((bottom* OR base) NEAR/3 (pyramid* OR piramid*)) OR ((resource*) NEAR/3 (constrain* OR scarc*)) OR bricoleur* OR bricolage* OR pro NEXT/1 poor* OR BoP OR jugaad or Jugaaṛ):ab,ti,kw) NOT ("conference abstract":kw OR Trial registry record:pt)

CINAHL

(MH "Telehealth+" OR MH "Digital Technology" OR TI(telehospital* OR tele-hospital* OR e-health* OR ehealth* OR mhealth* OR m-health* OR telehealth* OR tele-health* OR telemedic* OR tele-medic* OR telemonitor* OR tele-monitor* OR telecare OR tele-care OR tele-icu* OR tele-intensive-car* OR telephone-intervention* OR remote-consultat* OR telepresence* OR tele-presence* OR tele-referral* OR telereferral* OR teleconsultat* OR tele-consultat* OR tele-radiol* OR teleradiol* OR telepathol* OR tele-pathol* OR ((digital* OR remot* OR electronic* OR virtual* OR mobile) N3 (health* OR intervention* OR consult* OR diagno* OR medicine*)) OR health-information-technolog* OR health-IoT OR h-IoT OR uhealth OR telerehabilitat* OR tele-rehabilitat* OR ((remot*) N3 (monitor*)) OR ((smart*) N3 (health*)) OR ((health OR medical) N2 (internet-of-things)) OR ((digital* OR remot* OR electronic* OR virtual* OR mobile) N3 (distanc*) N3 (practice*))) OR AB(telehospital* OR tele-hospital* OR e-health* OR ehealth* OR mhealth* OR m-health* OR telehealth* OR tele-health* OR telemedic* OR tele-medic* OR telemonitor* OR tele-monitor* OR telecare OR tele-care OR tele-icu* OR tele-intensive-car* OR telephone-intervention* OR remote-consultat* OR telepresence* OR tele-presence* OR tele-referral* OR telereferral* OR teleconsultat* OR tele-consultat* OR tele-radiol* OR teleradiol* OR telepathol* OR tele-pathol* OR ((digital* OR remot* OR electronic* OR virtual* OR mobile) N3 (health* OR intervention* OR consult* OR diagno* OR medicine*)) OR health-information-technolog* OR health-IoT OR h-IoT OR uhealth OR telerehabilitat* OR tele-rehabilitat* OR ((remot*) N3 (monitor*)) OR ((smart*) N3 (health*)) OR ((health OR medical) N2 (internet-of-things)) OR ((digital* OR remot* OR electronic* OR virtual* OR mobile) N3 (distanc*) N3 (practice*)))) **AND** (TI(((inclusive* OR reverse* OR catalytic* OR lean OR cost* OR DIY OR sustain* OR grassroot* OR grass-root* OR bottom-up* OR bottomup* OR affordab* OR disruptive* OR lowcost*) N3 (innovat*)) OR bootstrap* OR boot-strap* OR frugal* OR ((bottom*

OR base) N3 (pyramid* OR piramid*)) OR ((resource*) N3 (constrain* OR scarc*)) OR bricoleur* OR bricolage* OR pro-poor* OR BoP OR jugaad or Jugaaṛ) OR AB(((inclusive* OR reverse* OR catalytic* OR lean OR cost* OR DIY OR sustain* OR grassroot* OR grass-root* OR bottom-up* OR bottomup* OR affordab* OR disruptive* OR lowcost*) N3 (innovat*)) OR bootstrap* OR boot-strap* OR frugal* OR ((bottom* OR base) N3 (pyramid* OR piramid*)) OR ((resource*) N3 (constrain* OR scarc*)) OR bricoleur* OR bricolage* OR pro-poor* OR BoP OR jugaad or Jugaaṛ)) **NOT** ((ZT "book") OR (ZT "book chapter") OR (ZT "commentary") OR (ZT "conference paper") OR (ZT "conference proceeding") OR (ZT "dissertation") OR (ZT "editorial") OR (ZT "letter") OR (ZT "letter to the editor") OR (ZT "newspaper"))

PsycINFO

(exp "Telemedicine"/ OR "Digital Technology"/ OR (telehospital* OR tele-hospital* OR e-health* OR ehealth* OR mhealth* OR m-health* OR telehealth* OR tele-health* OR telemedic* OR tele-medic* OR telemonitor* OR tele-monitor* OR telecare OR tele-care OR tele-icu* OR tele-intensive-car* OR telephone-intervention* OR remote-consultat* OR telepresence* OR tele-presence* OR tele-referral* OR telereferral* OR teleconsultat* OR tele-consultat* OR tele-radiol* OR teleradiol* OR telepathol* OR tele-pathol* OR ((digital* OR remot* OR electronic* OR virtual* OR mobile) ADJ3 (health* OR intervention* OR consult* OR diagno* OR medicine*)) OR health-information-technolog* OR health-IoT OR h-IoT OR uhealth OR telerehabilitat* OR tele-rehabilitat* OR ((remot*) ADJ3 (monitor*)) OR ((smart*) ADJ3 (health*)) OR ((health OR medical) ADJ2 (internet-of-things)) OR ((digital* OR remot* OR electronic* OR virtual* OR mobile) ADJ3 (distanc*) ADJ3 (practice*))).ab,ti.) **AND** ((((inclusive* OR reverse* OR catalytic* OR lean OR cost* OR DIY OR sustain* OR grassroot* OR grass-root* OR bottom-up* OR bottomup* OR affordab* OR disruptive* OR lowcost*) ADJ3 (innovat*)) OR bootstrap* OR boot-strap* OR frugal* OR ((bottom* OR base) ADJ3 (pyramid* OR piramid*)) OR ((resource*) ADJ3 (constrain* OR scarc*)) OR bricoleur* OR bricolage* OR pro-poor* OR BoP OR jugaad OR jugaar).ab,ti.) **NOT** ("Editorial".pt. OR "Comment".pt. OR "Letter".pt. OR "News".pt. OR "Congress".pt. OR "Meeting Abstract".pt. OR "Abstracts".pt. OR "Academic Dissertation".pt. OR "Published Erratum".pt. OR (dissertation abstract*).pt.)

SCOPUS

TITLE-ABS-KEY(telehospital* OR tele-hospital* OR e-health* OR ehealth* OR mhealth* OR m-health* OR telehealth* OR tele-health* OR telemedic* OR tele-medic* OR telemonitor* OR tele-monitor* OR telecare OR tele-care OR tele-icu* OR tele-intensive-car* OR telephone-intervention* OR remote-consultat* OR telepresence* OR tele-presence* OR tele-referral* OR telereferral* OR teleconsultat* OR tele-consultat* OR tele-radiol* OR teleradiol* OR telepathol* OR tele-pathol* OR ((digital* OR remot* OR electronic* OR virtual* OR mobile) W/3 (health* OR intervention* OR consult* OR diagno* OR medicine*)) OR health-information-technolog* OR health-IoT OR h-IoT OR uhealth OR telerehabilitat* OR tele-rehabilitat* OR ((remot*) W/3 (monitor*)) OR ((smart*) W/3 (health*)) OR ((health OR medical) W/2 (internet-of-things)) OR ((digital* OR remot* OR electronic* OR virtual* OR mobile) W/3 (distanc*) W/3 (practice*))) AND TITLE-ABS-KEY(((inclusive* OR reverse* OR catalytic* OR lean OR cost* OR DIY OR sustain* OR grassroot* OR grass-root* OR bottom-up* OR bottomup* OR affordab* OR disruptive* OR lowcost*) W/3 (innovat*)) OR bootstrap* OR boot-strap* OR frugal* OR ((bottom* OR base) W/3 (pyramid* OR piramid*)) OR ((resource*) W/3 (constrain* OR scarc*)) OR bricoleur* OR bricolage* OR pro-poor* OR BoP OR jugaad or Jugaaṛ) AND DOCTYPE(ar OR bk OR ch OR re)

Dimensions

(telehospital* OR tele-hospital* OR e-health* OR ehealth* OR mhealth* OR m-health* OR telehealth* OR tele-health* OR telemedic* OR tele-medic* OR telemonitor* OR tele-monitor* OR telecare OR tele-care OR tele-icu* OR tele-intensive-car* OR telephone-intervention* OR telepresence* OR tele-presence* OR tele-referral* OR telereferral* OR teleconsultat* OR teleradiol* OR tele-radiol* OR "digital health"~3 OR "digital healthcare"~3 OR "digital health care"~3 OR "digital intervention"~3 OR "digital consultation"~3 OR "digital diagnosis"~3 OR "digital medicine"~3 OR "remote health"~3 OR "remote healthcare"~3 OR "remote health care"~3 OR "remote intervention"~3 OR "remote consultation"~3 OR "remote diagnosis"~3 OR "remote medicine"~3 OR "electronic health"~3 OR "electronic healthcare"~3 OR "electronic health care"~3 OR "electronic intervention"~3 OR "electronic consultation"~3 OR "electronic diagnosis"~3 OR "electronic medicine"~3 OR "virtual health"~3 OR "virtual healthcare"~3 OR "virtual health care"~3 OR "virtual intervention"~3 OR "virtual consultation"~3 OR "virtual diagnosis"~3 OR "virtual medicine"~3 OR "mobile health"~3 OR "mobile healthcare"~3 OR "mobile health care"~3 OR "mobile intervention"~3 OR "mobile consultation"~3 OR "mobile diagnosis"~3 OR "mobile medicine"~3 OR health-information-technolog* OR health-IoT OR h-IoT OR uhealth OR "remote monitoring"~3 OR "remote monitor"~3 OR "smart health"~3 OR "smart healthcare"~3 OR "smart health care"~3 OR "health internet things"~3 OR "medical internet things"~3 OR "digital distance practice"~3 OR "digital distance practices"~3 OR "remote distance practice"~3 OR "remote distance practices"~3 OR "electronic distance practice"~3 OR "electronic distance practices"~3 OR "virtual distance practice"~3 OR "virtual distance practices"~3 OR "mobile distance practice"~3 OR "mobile distance practices"~3) **AND** ("inclusive innovation"~3 OR "inclusive innovations"~3 OR "reverse innovation"~3 OR "reverse innovations"~3 OR "catalytic innovation"~3 OR "catalytic innovations"~3 OR "lean innovation"~3 OR "lean innovations"~3 OR "cost innovation"~3 OR "cost innovations"~3 OR "DIY innovation"~3 OR "DIY innovations"~3 OR "sustainable innovation"~3 OR "sustainable innovations"~3 OR "grassroot innovation"~3 OR "grassroot innovations"~3 OR "grass root innovation"~3 OR "grass root innovations"~3 OR "bottomup innovation"~3 OR "bottomup innovations"~3 OR "bottom up innovation"~3 OR "bottom up innovations"~3 OR "affordable innovation"~3 OR "afordable innovations"~3 OR "disruptive innovation"~3 OR "disruptive innovations"~3 OR "lowcost innovation"~3 OR "lowcost innovations"~3 OR "low cost innovation"~3 OR "low cost innovations"~3 OR bootstrap* OR boot-strap* OR frugal* OR "bottom pyramide"~3 OR "bottom piramide"~3 OR "base pyramide"~3 OR "base piramide"~3 OR "resource constraint"~3 OR "resource constraining"~3 OR "resource scarce"~3 OR "resource scarcity"~3 OR "resource scarcities"~3 OR "resources constraint"~3 OR "resources constraining"~3 OR "resources scarce"~3 OR "resources scarcity"~3 OR "resources scarcities"~3 OR bricoleur* OR bricolage* OR pro-poor* OR BoP OR jugaad OR jugaar)

Manually selected

IEEE Xplore Digital Library

Max 25 zoektermen

Advanced search

Field *abstract*:

e-health OR e-healthcare OR ehealthcare OR mhealth OR mhealthcare OR m-health OR m-healthcare OR telehealth OR telehealthcare OR tele-health OR tele-healthcare OR telemedicine OR tele-medicine OR teleradiology OR tele-radiology OR digital-health*

AND

Field *abstract*:

frugal* OR innovation*

15 zoektermen

EconLIT

ABSTRACT(telehospital* OR tele-hospital* OR e-health* OR ehealth* OR mhealth* OR m-health* OR telehealth* OR tele-health* OR telemedic* OR tele-medic* OR telemonitor* OR tele-monitor* OR telecare OR tele-care OR tele-icu* OR tele-intensive-car* OR telephone-intervention* OR telepresence* OR tele-presence* OR tele-referral* OR telereferral* OR teleconsultat* OR teleradiol* OR tele-radiol* OR ((digital* OR remot* OR electronic* OR virtual* OR mobile) NEAR/3 (health* OR intervention* OR consult* OR diagno* OR medicine*)) OR health-information-technolog* OR health-IoT OR h-IoT OR uhealth OR ((remot*) NEAR/3 (monitor*)) OR ((smart*) NEAR/3 (health*)) OR ((health OR medical) NEAR/2 (internet-of-things)) OR ((digital* OR remot* OR electronic* OR virtual* OR mobile) NEAR/3 (distanc*) NEAR/3 (practice*))) AND ABSTRACT(((inclusive* OR reverse* OR catalytic* OR lean OR cost* OR DIY OR sustain* OR grassroot* OR grass-root* OR bottom-up* OR bottomup* OR affordab* OR disruptive* OR lowcost*) NEAR/3 (innovat*)) OR bootstrap* OR boot-strap* OR frugal* OR ((bottom* OR base) NEAR/3 (pyramid* OR piramid*)) OR ((resource*) NEAR/3 (constrain* OR scarc*)) OR bricoleur* OR bricolage* OR pro-poor* OR BoP OR jugaad OR jugaar)

InHTA database

(telehospital* OR tele-hospital* OR e-health* OR ehealth* OR mhealth* OR m-health* OR telehealth* OR tele-health* OR telemedic* OR tele-medic* OR telemonitor* OR tele-monitor* OR telecare OR tele-care OR tele-icu* OR tele-intensive-car* OR telephone-intervention* OR telepresence* OR tele-presence* OR tele-referral* OR telereferral* OR teleconsultat* OR teleradiol* OR tele-radiol* OR "digital health" OR "digital healthcare" OR "digital health care" OR "digital intervention" OR "digital consultation" OR "digital diagnosis" OR "digital medicine" OR "remote health" OR "remote healthcare" OR "remote health care" OR "remote intervention" OR "remote consultation" OR "remote diagnosis" OR "remote medicine" OR "electronic health" OR "electronic healthcare" OR "electronic health care" OR "electronic intervention" OR "electronic consultation" OR "electronic diagnosis" OR "electronic medicine" OR "virtual health" OR "virtual healthcare" OR "virtual health care" OR "virtual intervention" OR "virtual consultation" OR "virtual diagnosis" OR "virtual medicine" OR "mobile health" OR "mobile healthcare" OR "mobile health care" OR "mobile intervention" OR "mobile consultation" OR "mobile diagnosis" OR "mobile medicine" OR health-information-technolog* OR health-IoT OR h-IoT OR uhealth OR "remote monitoring" OR "remote monitor" OR "smart health" OR "smart healthcare" OR "smart health care" OR "health internet

things" OR "medical internet things" OR "digital distance practice" OR "digital distance practices" OR "remote distance practice" OR "remote distance practices" OR "electronic distance practice" OR "electronic distance practices" OR "virtual distance practice" OR "virtual distance practices" OR "mobile distance practice" OR "mobile distance practices") **AND** ("inclusive innovation" OR "inclusive innovations" OR "reverse innovation" OR "reverse innovations" OR "catalytic innovation" OR "catalytic innovations" OR "lean innovation" OR "lean innovations" OR "cost innovation" OR "cost innovations" OR "DIY innovation" OR "DIY innovations" OR "sustainable innovation" OR "sustainable innovations" OR "grassroot innovation" OR "grassroot innovations" OR "grass root innovation" OR "grass root innovations" OR "bottomup innovation" OR "bottomup innovations" OR "bottom up innovation" OR "bottom up innovations" OR "affordable innovation" OR "afordable innovations" OR "disruptive innovation" OR "disruptive innovations" OR "lowcost innovation" OR "lowcost innovations" OR "low cost innovation" OR "low cost innovations" OR bootstrap* OR boot-strap* OR frugal* OR "bottom pyramide" OR "bottom piramide" OR "base pyramide" OR "base piramide" OR "resource constraint" OR "resource constraining" OR "resource scarce" OR "resource scarcity" OR "resource scarcities" OR "resources constraint" OR "resources constraining" OR "resources scarce" OR "resources scarcity" OR "resources scarcities" OR bricoleur* OR bricolage* OR pro-poor* OR BoP OR jugaad OR jugaar)

LILACS

(telehospital OR telehospitals OR tele-hospital OR tele-hospitals OR e-health OR e-healthcare OR ehealthcare OR mhealth OR mhealthcare OR m-health OR m-healthcare OR telehealth OR telehealthcare OR tele-health OR tele-healthcare OR telemedicine OR telemedicines OR tele-medicine OR telemedicines OR telemonitoring OR tele-monitoring OR telecare OR tele-care OR tele-icu OR tele-intensive-care OR telephone-intervention OR telephone-interventions OR telepresence OR tele-presence OR tele-referral OR tele-referrals OR telereferral OR telereferrals OR teleconsultation OR teleconsultations OR teleradiology OR tele-radiology OR "digital health" OR "digital healthcare" OR "digital health care" OR "digital intervention" OR "digital consultation" OR "digital diagnosis" OR "digital medicine" OR "remote health" OR "remote healthcare" OR "remote health care" OR "remote intervention" OR "remote consultation" OR "remote diagnosis" OR "remote medicine" OR "electronic health" OR "electronic healthcare" OR "electronic health care" OR "electronic intervention" OR "electronic consultation" OR "electronic diagnosis" OR "electronic medicine" OR "virtual health" OR "virtual healthcare" OR "virtual health care" OR "virtual intervention" OR "virtual consultation" OR "virtual diagnosis" OR "virtual medicine" OR "mobile health" OR "mobile healthcare" OR "mobile health care" OR "mobile intervention" OR "mobile consultation" OR "mobile diagnosis" OR "mobile medicine" OR health-information-technolog* OR health-IoT OR h-IoT OR uhealth OR "remote monitoring" OR "remote monitor" OR "smart health" OR "smart healthcare" OR "smart health care" OR "health internet things" OR "medical internet things" OR "digital distance practice" OR "digital distance practices" OR "remote distance practice" OR "remote distance practices" OR "electronic distance practice" OR "electronic distance practices" OR "virtual distance practice" OR "virtual distance practices" OR "mobile distance practice" OR "mobile distance practices") AND ("inclusive innovation" OR "inclusive innovations" OR "reverse innovation" OR "reverse innovations" OR "catalytic innovation" OR "catalytic innovations" OR "lean innovation" OR "lean innovations" OR "cost innovation" OR "cost innovations" OR "DIY innovation" OR "DIY innovations" OR "sustainable innovation" OR "sustainable innovations" OR "grassroot innovation" OR "grassroot innovations" OR "grass root innovation" OR "grass root innovations" OR "bottomup innovation" OR "bottomup innovations" OR "bottom up innovation" OR "bottom up innovations" OR

"affordable innovation" OR "afordable innovations" OR "disruptive innovation" OR "disruptive innovations" OR "lowcost innovation" OR "lowcost innovations" OR "low cost innovation" OR "low cost innovations" OR bootstrap OR boot-strap OR frugal OR frugality OR "bottom pyramide" OR "bottom piramide" OR "base pyramide" OR "base piramide" OR "resource constraint" OR "resource constraining" OR "resource scarce" OR "resource scarcity" OR "resource scarcities" OR "resources constraint" OR "resources constraining" OR "resources scarce" OR "resources scarcity" OR "resources scarcities" OR bricoleur OR bricolage OR pro-poor OR BoP OR jugaad OR jugaar)
